# High-Glucose-Induced Injury to Proximal Tubules of the Renal System Is Alleviated by Netrin-1 Suppression of Akt/mTOR

**DOI:** 10.1155/2023/4193309

**Published:** 2023-11-21

**Authors:** Chenxiao Liu, Xingna Hu, Yun Zhao, Aijie Huang, Jiaqi Chen, Ting Lu, Mian Wu, Honghong Lu

**Affiliations:** Department of Endocrinology, The Affiliated Suzhou Hospital of Nanjing Medical University, Suzhou Municipal Hospital, Gusu School, Nanjing Medical University, 242 Guangji Road, Jiangsu 215008, China

## Abstract

The kidneys have a high level of Netrin-1 expression, which protects against some acute and chronic kidney disorders. However, it is yet unknown how Netrin-1 affects renal proximal tubule cells in diabetic nephropathy (DN) under pathological circumstances. Research has shown that autophagy protects the kidneys in animal models of renal disease. In this study, we looked at the probable autophagy regulation mechanism of Netrin-1 and its function in the pathogenesis of DN. We proved that in HK-2 cell, high blood sugar levels caused Netrin-1 to be downregulated, which then triggered the Akt/mTOR signaling pathway and enhanced cell death and actin cytoskeleton disruption. By adding Netrin-1 or an autophagy activator in vitro, these pathogenic alterations were reverted. Our results indicate that Netrin-1 stimulates autophagy by blocking the Akt/mTOR signaling pathway, which underlies high-glucose-induced malfunction of the renal proximal tubules. After HK-2 cells were incubated with Netrin-1 recombination protein and rapamycin under HG conditions for 24 h, the apoptosis was significantly reduced, as shown by the higher levels of Bcl-2, as well as lower levels of Bax and cleaved caspase-3 (*P* = 0.012, Cohen's *d* = 0.489, Glass's delta = 0.23, Hedges' *g* = 0.641). This study reveals that targeting Netrin-1-related signaling has therapeutic potential for DN and advances our knowledge of the processes operating in renal proximal tubules in DN.

## 1. Introduction

End-stage renal disease (ESRD) is the last stage (5th stage) of chronic kidney disease (CKD), with only 15% or less of the kidney functioning in filtration. ESRD, which is a consequence of diabetes, is largely brought on by diabetic nephropathy (DN). Nephrologists have shown a great deal of interest in the glomerular cell damage brought on by high glucose (HG) levels in DN; nevertheless, growing evidence suggests that renal tubule damage, specifically to the proximal tubule, occurs in DN [1]. In the aetiology of DN, progressive renal tubulointerstitial fibrosis plays a crucial role [2]. Intense scientific research has focused on the essential functions that renal proximal tubule damage and loss play in kidney disease [3].

A member of the netrin protein family, Netrin-1 is a secreted glycoprotein with a significant chemotropic role in axonal guidance [4]. Netrin-1 engages several different receptors to activate chemotropic responses and adhesive mechanisms in nonneural systems. Previous research has shown that the laminin-related secreted protein known as Netrin-1 is excreted in the urine of both mice and humans and is strongly increased following acute and chronic kidney injury. Furthermore, a study found that renal fibrogenesis is aided by aberrant Netrin-1 stimulation in the kidneys [5]. The mechanism underlying Netrin-1's impact on the renal proximal tubules in diseased situations is still unknown.

Animal models of kidney disorders have renoprotective effects due to autophagy, which is essential for cellular metabolism and organelle homeostasis [6]. Apoptosis results from impaired autophagy [7]. The Akt pathway is frequently abnormally activated and is linked to numerous disorders, including DN [8, 9], where it is crucial for mediating cell differentiation, cell cycle, metastasis, and apoptosis. When the Akt signaling pathway is activated, mTOR is phosphorylated, ultimately inhibiting autophagy. According to a recent study, Netrin-1 modulates autophagic activity by controlling lysosomal processes [10]. Exogenous Netrin-1 decreases autophagosome production and ultimately lowers autophagic activity, according to a different study [11]. Uncertainty exists on how the altered Akt/mTOR signaling and proximal tubule cell activity are affected by the reduced expression of Netrin-1 in DN.

To shed light on potential pathogenic causes and develop new therapeutic approaches for DN, the goal of this work was to examine the biological importance of Netrin-1 in high-glucose-triggered renal tubular cell dysfunction.

## 2. Materials and Methods

### 2.1. Cell Culture

The Shanghai Fuheng Biotechnology Co., Ltd. sold us the human renal proximal tubule (HK-2) (FH0228; Shanghai, China). 0.05 mg/mL bovine pituitary extract and 5 ng/mL epidermal growth factor were added to keratinocyte serum-free media (K-SFM; Invitrogen, Carlsbad, CA, USA), which was used to subculture the cells (EGF, Invitrogen). For 10-14 days, the cells were incubated in a CO_2_ environment at 37°C. Depending on the rate of cell growth, the medium was changed every day or every other day. Additionally, during 24 hours, HK-2 cells received either 5.5 mM (NG group) or 30 mM (HG group) glucose. Rapamycin (100 nmol/L), an inhibitor of mTOR, was bought from Selleckchem (S1039; Houston, Texas, USA). We bought rat recombinant Netrin-1 from R&D Systems, Inc. (6419-N1-025; Minneapolis, Minnesota, USA). For 24 hours, Netrin-1 (0 or 20 ng/mL) was applied to HK-2 cells.

### 2.2. Plasmid Transfection

Netrin-1-specific shRNA or scramble shRNA (Asia-Vector Biotechnology, Shanghai, China) was transiently transfected with HK-2 cells with Lipofectamine 2000 (Thermo Fisher Scientific, Waltham, USA), and the cells were subsequently cultured for 48 hours or the indicated periods. Total RNA was prepared and subjected to qPCR analysis. Total RNA was extracted using TRIzol reagent (Invitrogen) and reverse transcribed into complementary DNA (cDNA) using the PrimeScript RT Reagent Kit (Takara Bio, Kusatsu, Shiga, Japan). SYBR Premix Ex Taq II (Takara Bio) was used to detect gene expression changes by RT-qPCR. The mRNA levels of target genes were normalized to those of GAPDH. Primer sequences were as follows: Netrin-1: 5′-TGCAAGCCCTTCCACTACG-3′ (F) and 5′-TGTTGTGGCGACAGTTGAGG-3′ (R) and GAPDH: 5′-GGAGCGAGATCCCTCCAAAAT-3′ (F) and 5′-GGCTGTTGTCATACTTCTCATGG-3′ (R).

### 2.3. Western Blot Assay

Radioimmunoprecipitation assay lysis buffer (P0013B; Solarbio) was used to extract cell proteins, and 10% SDS-PAGE was used to separate them. Anti-Netrin-1 (20235-1-AP; Proteintech) and anti-cleaved caspase-3 were added after the membrane was transferred to PVDF (9664T; Cell Signaling Technology); anti-Bax (14796; Cell Signaling Technology), anti-Bcl-2 (AB1722; Millipore), anti-LC3 (12741S; Cell Signaling Technology), anti-P62 (5114S; Cell Signaling Technology), anti-Beclin-1 (3738S; Cell Signaling Technology), anti-p-Akt (4060S; Cell Signaling Technology), anti-Akt (4691; Cell Signaling Technology), anti-p-mTOR (ab109268; Abcam), anti-mTOR (ab134903; Abcam), anti-mouse IgG (HRP-linked, 7076; Cell Signaling Technology), anti-rabbit IgG (HRP-linked, 7074; Cell Signaling Technology), and anti-GAPDH (5174; Cell Signaling Technology) antibodies were applied. A method for enhanced chemiluminescence (ECL) detection was used to detect the membrane after PBS washing and secondary antibody incubation (Santa Cruz Biotechnology).

### 2.4. Analysis of Cell Apoptosis

After being digested with 0.25% trypsin (without EDTA) and centrifuged for five minutes at 1,000 rpm, the various groups of HK-2 cells were resuspended in PBS. After being stained with Annexin V-FITC (5 L) and PI (10 L) for 30 minutes in a binding buffer, the cells were removed. Then, using flow cytometry, the apoptosis rate was discovered (40302ES20; YEASEN).

### 2.5. Adenoviral Transfection of GFP-mRFP-LC3

GFP-mRFP-LC3-containing adenovirus vector was used to infect HK-2 cells (Asia-Vector Biotechnology, Shanghai, China). On brand-new media, HK-2 cells were cultured for a whole day. Then, HK-2 cells were observed using a confocal laser scanning microscope (FV1000; OLYMPUS) to track autophagy flux and gauge the quantity of yellow and green dots.

### 2.6. Score for Actin Cytoskeletal Disorder

Phalloidin-TRITC (G1401, Servicebio, Wuhan, China) was used to stain F-actin filaments in HK-2 cells so that they could be seen under a fluorescence microscope (Zeiss, Oberkochen, Germany). After being put into a 24-well plate, the cells were fixed in PBS containing 4% paraformaldehyde. The PBS solution with 0.5% Triton X-100 permeated the cell membrane. For cell structure labelling, DAPI (G1012; Servicebio) and phalloidin-TRITC (100 nM; G1401; Servicebio) were utilised. Using a grading system from a prior study, a semiquantitative analysis of actin cytoskeleton abnormality was carried out [12]. Phalloidin staining of F-actin revealed the disordered patches, which were classified as such.

### 2.7. Statistical Analysis

SEMs and means are used to express data. The means among more than two groups were analyzed using one-way ANOVA and the Bonferroni correction. When there were two groups, unpaired *t*-tests were performed to examine the data. For the independent sample *t*-test, we have utilised Cohen's *d* to calculate the mean difference between the two groups and then divide the original data by the pooled standard deviation. Cohen's *d* is the appropriate effect size measure if two groups have similar standard deviations and are of the same size. Glass's delta, which uses only the standard deviation of the control group, is an alternative measure if each group has a different standard deviation. Hedges' *g*, which provides a measure of effect size weighted according to the relative size of each sample, is an alternative where there are different sample sizes. The statistical evaluations used GraphPad Prism (v.8.1) programme. Representative results from each experiment, which were carried out at minimum three times, are displayed. The cutoff for statistical significance was *P* < 0.05.

## 3. Results

### 3.1. Netrin-1 Overexpression Reduced Hyperglycemia-Induced Apoptosis and Aberrant Actin Cytoskeleton

**Figure 1:**
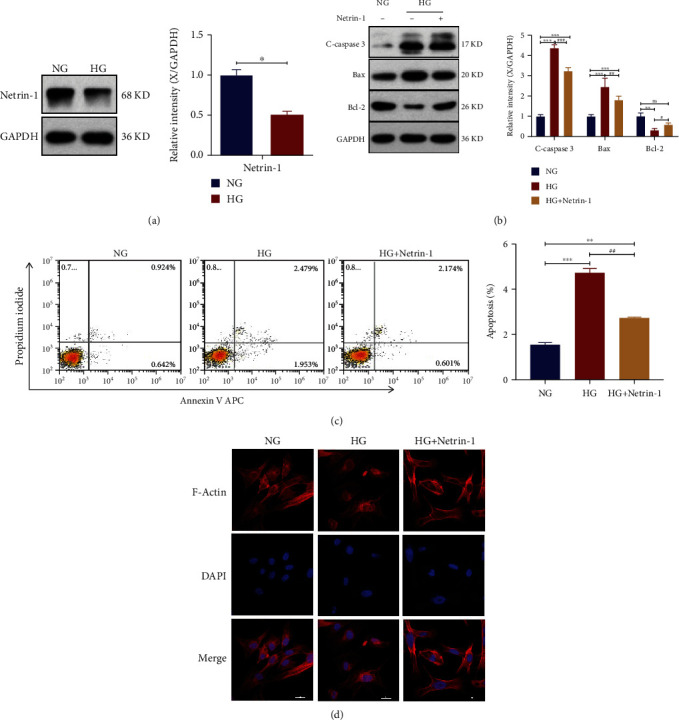
Overexpression of Netrin-1 ameliorated hyperglycemia-induced apoptosis and actin cytoskeleton derangement. (a) Western blotting analyses of Netrin-1 level in HK-2 cells under 30.0mMglucose treatment (HG group) compared with the control group (5.5mM glucose treatment, NG group); Band intensity of Netrin-1. (b) Western blotting analyses of Bcl-2, Bax and cleaved caspase-3 in HK-2 cells under the NG or HG. Equal protein loading was confirmed using an anti-GAPDH antibody; Band intensity of Bcl-2, Bax and cleaved caspase-3. (c) Flow cytometry analysis of apoptotic HK-2 cells under the NG (control), HG or HG plus treatment with recombinant Netrin-1 condition. (d) Fluorescein—phalloidin staining of actin fibers in cultured HK-2 cells. Actin cytoskeleton derangement was reduced in HK-2 cells under the HG plus treatment with recombinant Netrin-1 condition (scale bar = 20 *μ*m). All experiments were conducted in triplicate. Values are means ± SEMs. vs. LG group: ^∗^*P* < 0.05, ^∗∗^*P* < 0.01, and ^∗∗∗^*P* < 0.001. vs. HG group: ^∗^*P* < 0.05, ^∗∗^*P* < 0.01, ^∗∗∗^*P* < 0.001, ^#^*P* < 0.05, ^##^*P* < 0.01, ^###^*P* < 0.001.

HG treatment was shown to decrease Netrin-1 expression (Figure 1(a)). The development of DN is significantly influenced by the dysfunction of the renal proximal tubules. We found the function of Netrin-1 in apoptosis and the actin cell skeleton of HK-2 cells under HG circumstances to ascertain its impact on renal proximal tubules. As a signaling pathway that regulates cell apoptosis and survival, the Bcl-2/Bax/cleaved caspase-3 apoptotic signaling pathway has been implicated in many diseases including several nervous system diseases. Expression of proapoptotic proteins (cleaved caspase-3 and Bax) was noticeably upregulated in HG group while it was downregulated after exposure to recombinant Netrin-1 (Cohen's *d* = 0.632, Glass's delta = 0.5, Hedges' *g* = 0.607) (Figure 1(b)). It was discovered that Bcl-2, a crucial antiapoptotic protein, had the opposite impact. According to flow cytometry, the number of HK-2 cells that underwent apoptosis was much higher under the HG circumstances compared to the controls; however, adding Netrin-1 dramatically decreased cell death in comparison to cells under the HG circumstances only (Cohen's *d* = 0.478, Glass's delta = 0.611, Hedges' *g* = 0.225) (Figure 1(c)).

Netrin-1 recombination protein was added in the renal proximal tubule to examine whether it affects cytoskeletal reorganization. Treatment with HG dramatically increased the amount of disordered HK-2 cells and decreased the quantity of F-actin fibers, while overexpression of Netrin-1 had the opposite effects (Cohen's *d* = 0.469, Glass's delta = 0.331, Hedges' *g* = 0.346) (Figure 1(d)). These findings demonstrate the critical role of Netrin-1 in renal proximal tubule dysfunction in HG.

### 3.2. Netrin-1 Overexpression Promoted Autophagy and the Akt/mTOR Pathway

**Figure 2:**
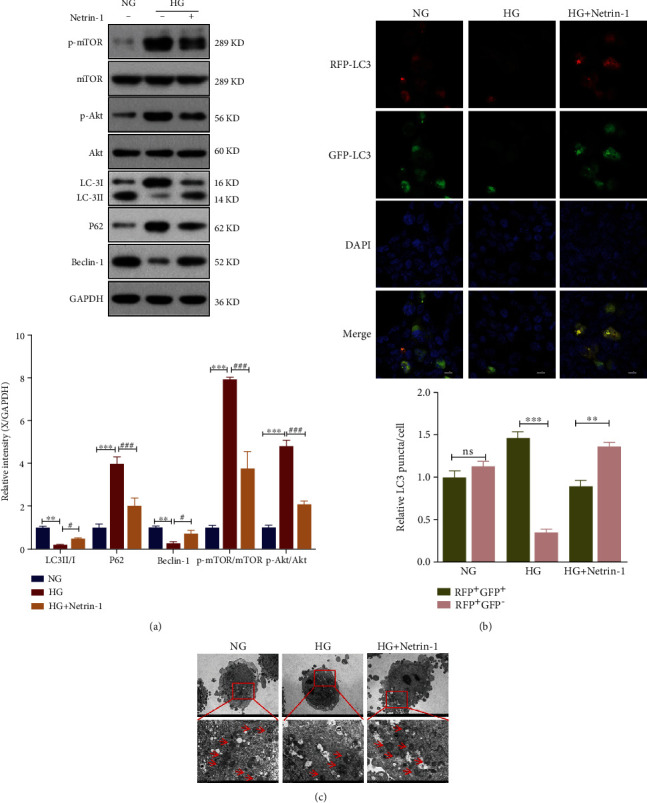
Overexpression of Netrin-1 mediated the Akt/mTOR pathway and induced autophagy. (a) Western blotting analyses of p-mTOR, mTOR, p-Akt, Akt, LC3, P62, and Beclin-1 in HK-2 cells under the NG or HG; Band intensities of p-mTOR/ mTOR, p-Akt/ Akt, LC-3Ⅱ/Ⅰ, P62 and Beclin-1. (b) Adenovirus encoding GFP-RFP-LC3 were transiently transfected into cells followed by treating with NG (5.5mM glucose), HG (30.0mM glucose) or HG+Netrin-1(30.0mM glucose+20ng/ml recombinant Netrin-1) for 24h, respectively. The intensity of GFP and RFP puncta was gauged by Image J program. Scale bars were 10 *μ*m. (c) Electron microscopy analyses number of autophagosome (original magnification and ~1,200x) in different group. Values are means ± SEMs. vs. LG group: ^∗^*P* < 0.05, ^∗∗^*P* < 0.01, and ^∗∗∗^*P* < 0.001. vs. HG group: ^#^*P* < 0.05, ^##^*P* < 0.01, and ^###^*P* < 0.001.

It was determined how HG circumstances affected the autophagy of HK-2 cells. To investigate the regulatory functions of Netrin-1 in autophagy, the expression levels of Beclin-1, P62, and LC3, three biological indicators of autophagic activity, were assessed in HG-treated HK-2 (Figure 2(a)). After receiving HG therapy, compared to the control category, Beclin-1 expression levels and the ratio of LC3-II/LC3-I expression levels both dropped. Additionally, after HG treatment, overexpression of Netrin-1 increased the ratio of LC3-II/LC3-I expression and the level of Beclin-1 expression, showing that HG treatment significantly decreased LC3 fluorescence intensity, whereas overexpression of Netrin-1 restored LC3 expression in HG-treated cells (Figure 2(b)). In HG circumstances, HK-2 cells showed a stable low autophagic activity. Cells that overexpressed Netrin-1, however, greatly increased autophagy (Figure 2(c)). According to these results, Netrin-1 therapy could be able to increase autophagy in HG-treated HK-2 cells.

Akt activates mTOR, a crucial protein in the control of autophagy, which thus prevents autophagy from occurring. A treatment with Netrin-1 resulted in lower ratios of p-Akt/Akt and p-mTOR/mTOR (Figure 2(a)). This result suggested that, in HG circumstances, Netrin-1 further suppressed the Akt/mTOR signaling pathway.

### 3.3. Decreased Netrin-1 Expression Reduced Autophagy

**Figure 3:**
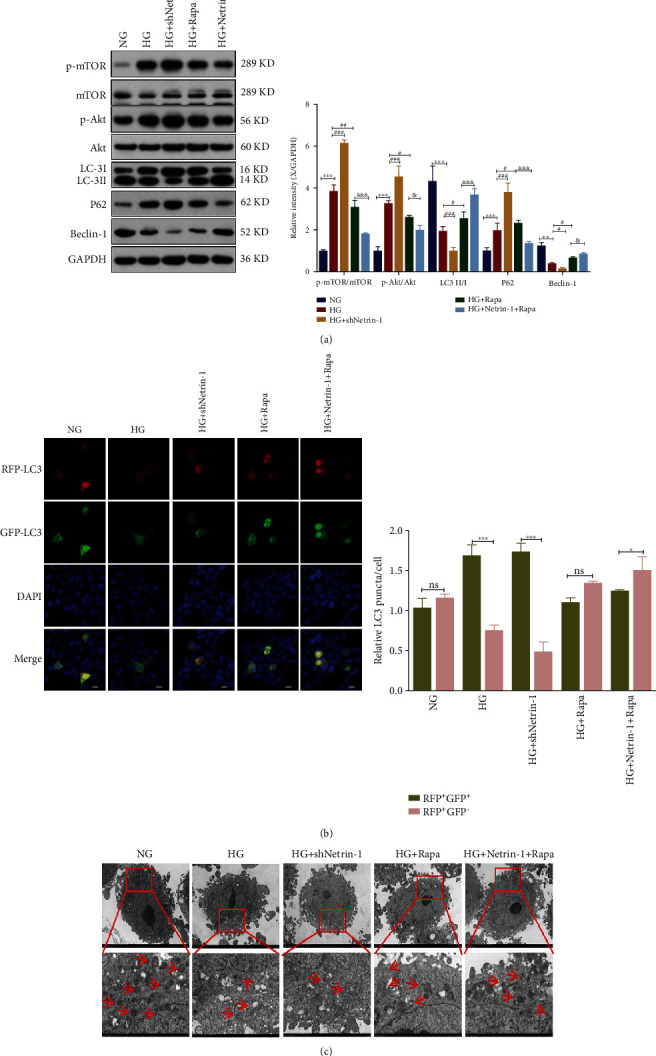
Decreased Netrin-1 expression reduced autophagy.(a) Western blotting analyses of p-mTOR, mTOR, p-Akt, Akt, LC3, P62, and Beclin-1 in HK-2 cells under the NG, HG, HG plus treatment with a plasmid expressing an shRNA against Netrin-1 (shNetrin-1), HG plus rapamycin (a mTOR inhibitor, 100nmol/L) and HG+Netrin-1+Rapa; Band intensities of p-mTOR/ mTOR, p-Akt/ Akt, LC-3Ⅱ/Ⅰ, P62 and Beclin-1. (b) Adenovirus encoding GFP-RFP-LC3 were transiently transfected into cells followed by treating with above treatment for 24h, respectively. The intensity of GFP and RFP puncta was gauged by Image J program. Scale bars were 10 *μ*m. (c) Electron microscopy analyses number of autophagosome (original magnification and ~1,200x) in different group. Values are means ± SEMs. ^∗^*P* < 0.05, ^∗∗^*P* < 0.01, and ^∗∗∗^*P* < 0.001. vs. HG group: ^#^*P* < 0.05, ^##^*P* < 0.01, and ^###^*P* < 0.001. vs.HG+Rapa group: ^&^*P* < 0.05, ^&&^*P* < 0.01, and ^&&&^*P* < 0.001.

We used shRNA to silence the expression of Netrin-1 in HK-2 cells under high-glucose environment and used rapamycin (Rapa) to induce autophagy on the basis of high glucose, combined with recombinant Netrin-1, and then detected apoptosis by flow cytometry and Western blot. The expression of autophagy-related proteins was detected, and the autophagy was observed by immunofluorescence double labeling and electron microscopy. GFP-LC3 varied with acidity, but when autophagy was activated to form autolysosomes, RFP-LC3 showed enhanced red dots. GFP and RFP dots represent autophagosomes and autophagolysosomes, respectively, and are represented as yellow dots when combined. When the number of red dots is significantly more than that of green dots, we believe that autophagy flow is relatively uninhibited. The results of WB showed that compared with the HG group, the expressions of LC3 and Beclin-1 in the HG+shNetrin-1 group were further decreased, the accumulation of autophagy substrate P62 was significantly increased, and the expressions of p-mTOR/mTOR and p-Akt/Akt were also significantly increased. The above results were reversed after the addition of rapamycin. Compared with the HG group, the expressions of LC3II/LC3I and Beclin-1 were increased after HG+ rapamycin treatment (*P* < 0.05), while the expression level of P62 was decreased (*P* < 0.05), indicating that autophagy of HK-2 cells was enhanced, and the expressions of P-mtor /mTOR and P-Akt/Akt were significantly decreased. This indicated that the Akt/mTOR pathway was inhibited (Figure 3(a)), and the gray-scale analysis was consistent. Confocal microscopy showed that, similar to hyperglycemia, hyperglycemia combined with shNetrin-1 significantly increased the proportion of RFP+GFP+ cells (*P* < 0.001), while the proportion of RFP+GFP- cells showing red fluorescence increased after the addition of autophagy activator Rapa on the basis of hyperglycemia alone. However, there was no statistical difference compared with RFP+GFP+ cells, and the proportion of RFP+GFP- cells exhibiting red fluorescence significantly increased under high-glucose environment combined with overexpression of Netrin-1 and addition of Rapa (*P* < 0.001) (Figure 3(b)). Then, the changes of intracellular autophagosomes were observed by electron microscopy. The results showed that the number of autophagosomes in the HG+shNetrin-1 group significantly decreased, and the number of autophagosomes increased after rapamycin treatment and further increased after overexpression of Netrin-1 (Figure 3(c)).

### 3.4. Rapamycin Decreases High-Glucose Injury in HK-2 Cells

**Figure 4:**
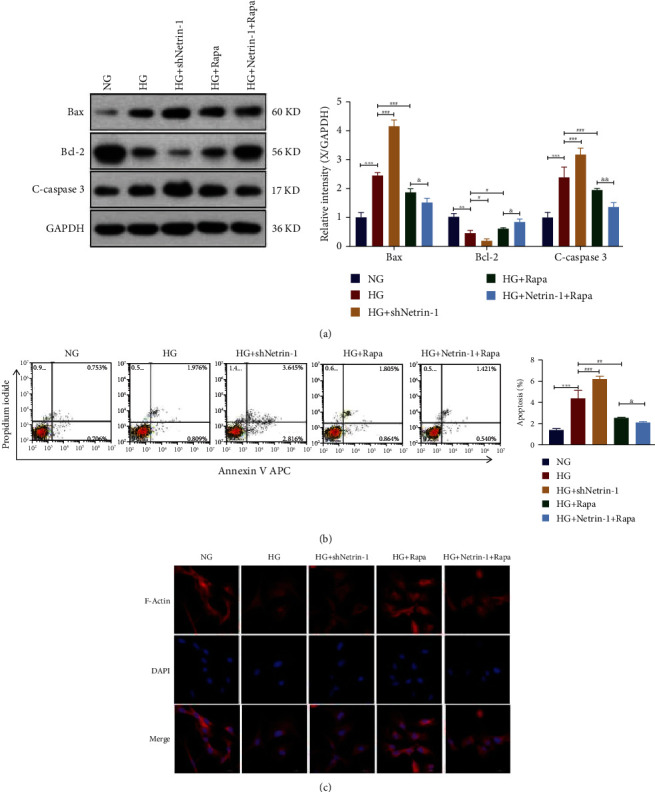
Rapamycin decreases high-glucose injury in HK-2 cells.(a)Flow cytometry and quantification to assay apoptosis of HK-2 cells under the NG, HG, HG plus treatment with a plasmid expressing an shRNA against Netrin-1 (shNetrin-1), HG plus rapamycin (a mTOR inhibitor) and HG+Netrin-1+Rapa. (b) Western blotting analyses of Bcl-2, Bax and cleaved caspase-3 in HK-2 cells. Equal protein loading was confirmed using an anti-GAPDH antibody; Band intensity of Bcl-2, Bax and cleaved caspase-3. (c) Fluorescein—phalloidin staining of actin fibers with indicated treatments and transfections (scale bar = 20 *μ*m). Values are means ± SEMs. ^∗^*P* < 0.05, ^∗∗^*P* < 0.01, and ^∗∗∗^*P* < 0.001. vs. HG group: ^∗^*P* < 0.05, ^∗∗^*P* < 0.01, and ^∗∗∗^*P* < 0.001. vs.HG+Rapa group: ^&^*P* < 0.05, ^&&^*P* < 0.01, and ^&&&^*P* < 0.001.

It was hypothesized that HK-2 cells secreted Netrin-1 to activate autophagy, thereby inhibiting apoptosis and actin cytoskeleton derangement. To test this hypothesis, Netrin-1 synthesis was knocked down in differentiated HK-2 cells using plasmid-mediated shRNA interference or by adding rapamycin. HK-2 cells incubated with shNetrin-1 under HG conditions for 24 h showed increased apoptosis (*P* < 0.001, Figure 4(a)), as indicated by the decreased levels of Bcl-2 and increased levels of cleaved caspase-3 and Bax. These effects were nullified by the addition of rapamycin (Figure 4(b)), as was increased Netrin-1 induced cytoskeletal derangement (Figure 4(c)).

## 4. Discussion

Under HG circumstances, Netrin-1 expression in renal proximal tubule cells was considerably reduced. Additionally, in HG circumstances, it triggers proximal tubule cell death and actin cytoskeleton derangement. Additionally, these effects were -worsen by utilising a shRNA to suppress Netrin-1. Finally, Netrin-1-induced protection of proximal tubule cells may be due to decreased apoptosis and the promotion of autophagy.

Under HG circumstances and in diabetic patients, there has been an increase in renal tubular epithelial cell apoptosis [13]. Dysfunction of the proximal tubule due to apoptosis characterizes the early stages of DN [14]. To identify new therapeutic strategies, we first examined the protective effects of Netrin-1 against proximal tubule cell apoptosis in diabetic kidneys. Our study provides evidence that HG treatment reduced Netrin-1 expression. Apoptosis in HK-2 cells is further triggered by Netrin-1 downregulation. The concentrations of Bcl-2, cleaved caspase-3, and Bax were measured to detect proximal tubule apoptosis accurately. This process can be blocked by the upregulation of Netrin-1 expression. Conversely, the expression of the autophagy activator rapamycin reversed the effects of HG's suppression of apoptosis in the shNetrin-1 transfection group.

Cell movement depends on the rearrangement of the cytoskeleton, which is a process that has been extensively investigated [12, 15]. Related proteins to this process include actin stress fibers, microtubules, and microfilaments. Our research revealed that overexpressing Netrin-1 in HK-2 cells inhibited actin reorganization and that Netrin-1 potentially alters proximal tubular epithelial cell morphology by activating autophagy under HG conditions.

PI3K/Akt/mTOR signaling pathway plays a vital role in the regulation of cell survival, growth, proliferation, angiogenesis, transcription, translation, and metabolism. Akt/mTOR signaling is stimulated by HG and is linked to proximal tubular injury and autophagy suppression in DN, according to earlier research [16]. In order to maintain intracellular lysosomal homeostasis in diabetic patients, autophagy is crucial [17]. Because DN is prevented in its early stages by the mTOR inhibitor rapamycin, diabetic kidney morphological and functional abnormalities are decreased [18]. These findings suggest that Akt/mTOR signaling is essential for regulating autophagy and proximal tubular injury and that targeting this signaling pathway is a promising way to ameliorate DN progression. In renal proximal tubular epithelial cells, a prior study revealed that Netrin-1 might activate the Akt/mTOR pathway [19, 20].

In neural growth cones, Netrin-1 inhibits the phosphorylated/activated versions of Akt and mTOR [21]. Similar to this, our research showed that proximal tubular epithelial cells treated with HG increased phosphorylation of Akt and mTOR. Akt and mTOR activities were, however, markedly suppressed by Netrin-1 overexpression. Incubation with rapamycin exacerbated a increase in LC3II brought on by decreased Netrin-1 expression, indicating that Netrin-1 triggers Akt/mTOR-mediated autophagy.

In conclusion, decreased Netrin-1 expression under HG conditions results in renal proximal tubule cell apoptosis and actin cytoskeletal derangement. By inhibiting the Akt/mTOR signaling pathway and protecting cells from apoptosis and actin cytoskeletal disorder, upregulation of Netrin-1 led to autophagy activity in HK-2 cells. These discoveries shed new light on the processes of autophagy and apoptosis. As a result, techniques based on Netrin-1 may be used to treat DN. Additionally, the crucial role that Netrin-1 plays in DN suggests that manipulating pathways connected to Netrin-1 is likely to be successful.

## Data Availability

We will not share our data because it involves people's privacy and needs more research.
